# Investigation of a new signalling pathway linking deltaD to ciliogenesis - the role of rabconnectin3

**DOI:** 10.1186/2046-2530-1-S1-P50

**Published:** 2012-11-16

**Authors:** B Tavares, A Vaz, P Pintado, SS Lopes

**Affiliations:** 1 Faculty of Medical Sciences, CEDOC, Portugal

## 

Recently, using the zebrafish mutant for the *deltaD* gene (*after eight* or *aei-/-*), our group has showed that Notch signaling was involved in the control of cilia length in the cells of the fish laterality organ, the Kupffer’s Vesicle (KV) (Lopes el al. 2010). Further research based on microarray screening allowed the discovery of several genes with differential expression in KV cells from *aei-/-* mutants compared to WT embryos. One of these genes, *rabconnectin3* or *rc3* (*ENSDARG00000091293*) was found to be severely downregulated. Homologs of this gene have recently been associated with Notch signaling in *Drosophila* and mammalian cells (Yan et al. 2009; Sethi et al. 2010, respectively) through the regulation of the V-ATPase activity. Furthermore, the activity of this pump has also been associated with the ciliogenesis in the KV (Chen et al. 2011). Using a Morpholino against *rc3*, we caused a decrease in the cilia length of the KV. We propose that the decrease in the cilia size present in the KV of the *aei-/-* mutants is caused by the deregulation of the transcription of genes such as *rc3* and not necessarily by the disruption of the Notch signaling.

http://www.cedoc.org/
